# Influencing factors of hospitalization costs for patients with subsequent osteoporotic hip fractures: multilevel model analysis by fracture episode

**DOI:** 10.3389/fendo.2026.1853745

**Published:** 2026-08-24

**Authors:** Jing Yan, Bifan Zhu, Yan Zhang, Xiaoting Yang, Nan Zhang, Bao Liu, Chunlin Jin

**Affiliations:** 1 Shanghai Health Development Research Center (Shanghai Medical Information Center), Shanghai, China; 2 Department of Health Economics, School of Public Health, Fudan University, Shanghai, China; 3 Department of Endocrinology, Shanghai Baoshan Hospital of Integrated Traditional Chinese and Western Medicine, Shanghai, China; 4 Department of Physical Medicine and Rehabilitation, Shanghai Putuo People’s Hospital, Shanghai, China; 5 Key Laboratory of Health Technology Assessment (Fudan University), National Health Commission, Shanghai, China

**Keywords:** hospitalization, hospital-level clustering, inpatient costs, osteoporotic hip fracture, refracture

## Abstract

**Background:**

Osteoporotic hip fractures often require immediate inpatient care and impose a substantial financial burden. Previous studies mainly focused on overall costs for patients with refractures, without disaggregating hospitalization costs by episode, leaving the determinants of first- and second-admission costs unclear in this subgroup. This study aimed to examine factors associated with hospitalization costs for each episode and to identify factors characterizing cost differences between the two hip-fracture hospitalizations in patients with subsequent osteoporotic hip fractures.

**Methods:**

Data were obtained from the Shanghai Health Statistics Center, covering all medical institutions in Shanghai, China, from January 1, 2013, to December 31, 2022. Multilevel models were used to identify factors related to hospitalization costs for the first and second fractures. Hospitalization variations and cost differences between the two fracture episodes were examined, and multivariable quantile regression was used to explore factors associated with cost differences.

**Results:**

Of 43,963 patients hospitalized for their first osteoporotic hip fracture, 1,152 experienced a subsequent hip fracture and required rehospitalization during the study period. At the first fracture, most patients were female (78.73%) and aged ≥80 years (57.47%). Grade-A tertiary hospitals represented 27.59% (*n* = 24) and 24.04% (*n* = 25) of treating hospitals for the first and second fractures, respectively, but admitted 55.64% (*n* = 641) and 51.39% (*n* = 592) of patients. For both episodes, fourth-degree surgery had the highest median costs ($9,787.15 and $9,726.55, respectively), whereas unoperated patients had the lowest ($1,147.75 and $1,133.36). Costs rose substantially with longer length of stay (LOS), reaching $11,197.72 and $11,729.95 for stays of ≥31 days. Null multilevel models of hospitalization costs revealed ICCs of 0.333 and 0.338 for the first and second fractures, respectively, both indicating significant hospital-level variance (all *P* < 0.001). Random-intercept models showed that grade-A tertiary hospitals showed higher adjusted costs than grade-B secondary hospitals for both fractures, and that higher surgery levels and longer LOS were positively associated with costs across both episodes (all *P* < 0.001). For the second fracture, an interval of 1 to 1.5 years between hospitalizations was associated with significantly lower costs compared with an interval of ≤1 year (exp(*β*) = 0.899; 95%*CI*, 0.824 to 0.980; *P* < 0.05). Quantile regression showed that a higher hospital level and longer LOS during the second hospitalization were associated with increased relative costs across quantiles (all *P* < 0.05). Conversely, a higher surgery level in the second hospitalization was associated with reduced relative costs at the 25th and 50th percentiles (all *P* < 0.05), indicating heterogeneous associations across different quantiles of the cost distribution.

**Conclusions:**

Among patients with subsequent osteoporotic hip fractures, grade-A tertiary hospitals were associated with higher adjusted costs than grade-B secondary hospitals for both fracture episodes, and inpatient costs increased progressively with higher surgery levels and longer LOS. Between-episode variations in hospital level, surgery level, and LOS exhibited heterogeneous effects on cost differences across quantiles. These findings highlight the need for episode-specific cost-control strategies that also account for variations between the two hospitalizations.

## Introduction

Osteoporotic fractures are serious injuries that usually require hospital admission, as they significantly decrease patients’ independence, functional ability, and quality of life (1–5). Compared with other fractures, osteoporotic hip fractures are associated with higher mortality, postoperative complications, and medical expenses (6). As a populous country, China is experiencing a rapidly aging population, with the proportion of the population over 65 years old expected to reach about 30% by 2050 (7). The crude incidence of hip fractures is expected to increase quickly in China as the country’s population ages (8). By 2050, it is predicted that there will be 4.5 million hip fracture cases worldwide, with Asia, particularly China, probably accounting for nearly half of these cases (9).

Patients with hip fractures often need complex surgeries like hip replacements, and acute inpatient treatment accounts for a large part of their medical costs (10–12), placing a significant economic burden on the healthcare system. Several studies confirmed that total hospitalizations for patients with osteoporotic hip fractures have shown a sustained upward trend, and at the same time, inpatient costs have increased accordingly (13–15). Subsequent fractures not only have a high risk of occurrence but also lead to significantly increased mortality rates, poorer functional outcomes, and higher medical costs compared to patients without subsequent fractures (16–18). Existing literature has predominantly focused on overall healthcare costs among hip fracture patients or cumulative costs associated with refractures (19–22), rather than analyzing hospitalization costs attributable to each fracture episode. Consequently, factors associated with hospitalization costs for the first and second episodes remain unclear. To enable separate examination of factors associated with inpatient costs for each fracture episode, the present study focused on patients with a subsequent hip fracture, analyzing episode-specific cost-associated factors.

Previous studies have noted that due to the complexity of treatment and poor prognosis of hip fractures (19), patients often choose grade-A tertiary hospitals for treatment because of their higher standards, more advanced medical equipment, and better infrastructure (21, 22), which may lead to clustering of patients at specific hospitals. Therefore, it is essential to account for the random effects of medical institutions (level 2) on patient-level (level 1) hospitalization costs to better identify the factors impacting these costs. However, most studies use a single-level model to analyze hospitalization costs (20–23), which may not be the optimal statistical model. Although a recent study used a multilevel model to examine costs associated with hip fracture patients, it focused on hospital organizational factors and did not account for patient-level factors, which could have led to an overestimation of the influence of hospital-level variables (24).

Exploring the individual and institutional factors that influence hospitalization costs among patients with subsequent osteoporotic hip fractures may offer insights into strategies to reduce individual economic burdens and improve resource allocation. This study, therefore, examined, for each episode separately, the inpatient costs of the first and second osteoporotic hip fractures among patients who experienced refractures, and used distinct multilevel models to analyze the determinants of the hospitalization costs for the first and second fractures, respectively. Furthermore, we also investigated the determinants of cost differences between the two hip fracture hospitalizations by multivariable quantile regression.

## Subjects and methods

### Data source

In this study, data were extracted from the front page of inpatient medical records through the hospital information system and compiled by the Shanghai Health Statistics Center. The variables included key patient information, such as gender, date of birth, insurance type, occupation, marital status, admission and discharge dates, disease code, surgery level, method of discharge, and detailed cost information. The data covered all medical institutions in Shanghai, China, from January 1, 2013, to December 31, 2022.

### Study design

All patient records with hospitalization during the pre-index period (January 1, 2013, to December 31, 2016) were excluded to ensure that only newly diagnosed cases of osteoporotic hip fractures were included. The subject identification period for this study was from January 1, 2017, to December 31, 2020. The date of the first hospitalization record during this period was designated as the index date; patients admitted after this period were excluded. Additionally, the observation window after the index hospitalization was set to ensure at least 2 years of available hospitalization records. The observation period ended at discharge from a second hospitalization for osteoporotic hip fracture or at the end of the study period (December 31, 2022), whichever came first.

### Study population

**Figure 1:**
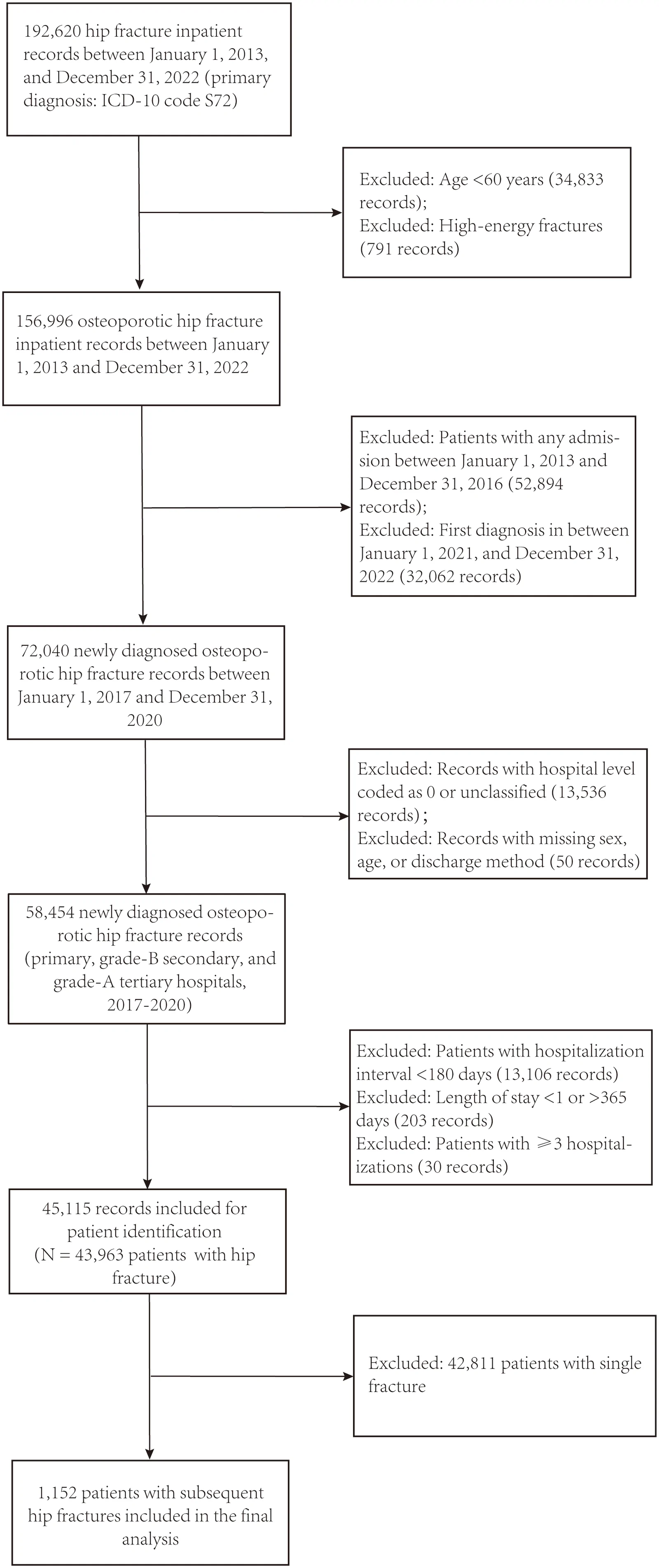
Patient flow chart.

Hip fracture was identified using the inpatient principal diagnosis (ICD code S72). Osteoporosis is often clinically silent, and many patients remain undiagnosed until their first fracture event. Restricting inclusion to patients with a documented osteoporosis diagnosis would therefore introduce severe selection bias and exclude a substantial number of true cases. Consistent with the Guidelines for the Diagnosis and Treatment of Primary Osteoporosis (2022), an osteoporotic fracture is defined as one caused by minor trauma; a low-energy hip fracture in an older adult is clinically diagnostic of severe osteoporosis, independent of bone mineral density testing (25). Multiple studies have also demonstrated that hip fracture is the hallmark site of osteoporotic fracture, and that a low-energy hip fracture signals advanced underlying bone fragility (26–28). Osteoporosis is strongly associated with age; the majority of hip fractures in individuals aged 60 years or older are osteoporotic fragility fractures (29, 30). Therefore, this study identified osteoporotic hip fracture patients using an operational definition based on (1) principal discharge diagnosis of ICD-10 code S72 (fracture of femur), (2) age ≥60 years, and (3) exclusion of high-energy trauma (e.g., traffic accidents, falls from height, polytrauma) through text screening of the pathologic diagnosis (**Figure 1**).

This study included 1,152 patients with osteoporotic hip fractures who experienced a subsequent hip fracture between January 1, 2017, and December 31, 2022. According to the literature, refractures at the same site require a minimum healing time of six months (10, 31, 32). Additionally, patients with hip fractures typically experience reduced mobility afterward, which lowers the risk of a subsequent fracture. Since this study focused on patients with subsequent osteoporotic hip fractures, a 6-month washout period was implemented to avoid double-counting and misclassification of rehabilitation hospitalizations as refractures. A second fracture was identified only if the interval between hospitalizations for osteoporotic hip fractures was at least 180 days.

### Derived variables

The hospitalization interval was defined as the number of days between the discharge date of the last hospitalization and the admission date of the next hospitalization for hip fracture, categorized as ≤1 year, 1~1.5 years, 1.5~2 years, and ≥2 years. Discharge status was dichotomized as regular discharge (discharge on medical advice) and non-regular discharge (transfer to another hospital, transfer to community health services/township health centers, unprescribed transfers, death, or other) due to small numbers in the original categories.

### Costing

The costs in this study were defined as the total direct medical expenses incurred during hospitalization for osteoporotic hip fracture, as recorded on the front page of the inpatient medical records. Cost difference was calculated as the total direct medical expenses of the subsequent hospitalization minus those of the index hospitalization. These costs comprised all payment sources (basic medical insurance, out-of-pocket, and others). Inpatient costs were adjusted to 2022 price levels using the chain-linked healthcare Consumer Price Index (CPI) for Shanghai published in the Shanghai Statistical Yearbook (**Supplementary Table S1**). Based on the RMB-to-USD exchange rate at the end of 2022, the CPI-adjusted costs were converted from Chinese yuan (CNY) to US dollars (USD). In 2022, the CNY-to-USD exchange rate was 6.9646. This rate was obtained from the China Foreign Exchange Trade System, authorized by the People’s Bank of China. For local policy relevance, all cost-related results are also presented in CNY in **Supplementary Tables S2**–**S4**.

### Hospital classification

Hospitals were categorized into three levels based on several factors, including hospital size, medical infrastructure, and technical capacity (33, 34). Primary hospitals are grassroots institutions that provide care directly to communities. Their main role is to provide basic medical services to the population, and they are equipped with fewer than 100 beds. Grade-B secondary hospitals are regional facilities serving multiple communities. Their primary function is to handle patients who cannot be managed by primary hospitals—those with complex conditions requiring specialized treatment or advanced equipment—and they have between 101 and 500 beds. Grade-A tertiary hospitals operate across regions, provinces, and municipalities, and even serve patients nationwide. They focus on delivering specialized medical services, accepting referrals from secondary hospitals, and treating critical and intractable diseases, with more than 501 beds.

Depending on their type, hospitals were classified as comprehensive or non-comprehensive (35). Comprehensive hospitals provide integrated, continuous care across multiple clinical departments (including internal medicine, surgery, obstetrics & gynecology, and pediatrics). In contrast, non-comprehensive hospitals lack this multidisciplinary structure and do not deliver integrated medical services. In this study, non-comprehensive hospitals include traditional Chinese medicine hospitals, integrative Chinese-Western medicine hospitals, specialty hospitals, nursing homes, convalescent centers, internet hospitals, community health service centers, maternal and child health facilities, and specialized disease prevention and treatment centers.

Additionally, hospitals were categorized as either for-profit or non-profit hospitals by institutional governance (36). Non-profit hospitals are operated for the public benefit, with revenues restricted to covering medical costs and institutional development; the distribution of surpluses to investors is prohibited, and they follow government-regulated pricing with tax benefits. These hospitals may be government-operated or privately established. In contrast, for-profit hospitals are mainly established to generate returns for owners or shareholders.

### Surgical classification

Surgical procedures were classified into four levels according to risk, complexity, technical difficulty, and resource consumption: level 1 (low risk, simple, low technical difficulty), level 2 (moderate risk, moderately complex, moderate technical difficulty), level 3 (relatively high risk, relatively complex, considerable difficulty, substantial resource consumption), and level 4 (high risk, complex, high difficulty, extensive resource consumption or major ethical risk) (37).

### Statistical analysis

Given the hierarchical nesting of patients within hospitals, a two-level random-intercept model was employed, with patients as level-1 units and hospitals as level-2 units. Because inpatient costs were heavily right-skewed, the natural logarithm of hospitalization costs was used as the dependent variable in the two-level multilevel model. Coefficients (*β*) from the log-scale model were back-transformed by exponentiation (exp(*β*)) to obtain multiplicative effects on the original cost scale. Model building followed a stepwise procedure guided by likelihood-ratio (LR) tests and information criteria (Akaike information criterion (AIC) and Bayesian information criterion (BIC)).

The null model was first fitted to quantify between-hospital variance:


$$Y_{ij}=\beta_{0}+u_{0j}+e_{0ij}$$

where β_0_ is the overall mean log cost, u_0_*_j_* ~ N(0, $\sigma_{u_{0}}^{2}$) is the hospital-level random intercept, and *e*_0_*_ij_*~ N(0, $\sigma_{e_{0}}^{2}$) is the patient-level residual.

The intra-class correlation (ICC) is calculated as:


$$ICC=\frac{\sigma_{u_{0}}^{2}}{\sigma_{u_{0}}^{2}+\sigma_{e_{0}}^{2}}$$

where $\sigma_{u_{0}}^{2}$ is the between-hospital variance and $\sigma_{e_{0}}^{2}$ is the within-hospital (patient-level) variance. If the ICC approaches 0, the difference between the two levels is slight; if the ICC approaches 1, the difference between the two levels is large. The higher the ICC value, the greater the proportion of the total variation in the dependent variable attributable to differences between groups. An ICC over 10% indicates that the data has a hierarchical nested structure.

All candidate level-1 covariates were entered simultaneously; gender, marital status, and work were sequentially removed because their exclusion did not significantly degrade fit (all LR *P* > 0.05). At level-2, the block of hospital-level variables significantly improved the model beyond the Level-1-only specification (for the first fracture: LR *χ*² = 9.94, df = 4, *P* = 0.042; for the second fracture: LR *χ*² = 28.34, df = 4, *P* < 0.001). Within this block, hospital type and hospital management type were non-significant and removed, leaving hospital level as the sole level-2 predictor in the final model, which achieved the lowest AIC and a competitive BIC (**Supplementary Tables S5**, **S6**).

For each multilevel model, multicollinearity among fixed-effect covariates was examined using the variance inflation factor (VIF) and condition index, with thresholds of 10 and 30, respectively, to ensure the stability of coefficient estimates (**Supplementary Table S7**). Multilevel model assumptions were subsequently verified: level-1 residual normality and homoscedasticity were assessed via Q-Q plots and residuals-versus-fitted plots, and level-2 random effect normality was confirmed via empirical Bayes prediction Q-Q plots and histograms (**Supplementary Figure S1**).

Model diagnostics were performed to compare the log-linear mixed model with a generalized linear mixed model (GLMM) assuming a gamma distribution and log link (**Supplementary Figure S2**). Sensitivity analyses using the GLMM specification confirmed the robustness of the findings (**Supplementary Table S8**).

To examine the determinants of cost differences between the two hospitalizations, multivariable quantile regression was used at the 25th, 50th, and 75th percentiles. This approach is robust to the skewness and heteroscedasticity of medical cost data and allows covariate effects to vary across the distribution. Bootstrap standard errors (400 replications) were used to estimate 95% *CI*. All models were adjusted for gender, age group, work, marital status, insurance type, discharge method, and interval between hospitalizations.

Continuous variables were compared using the Mann-Whitney U test for two independent groups or the Kruskal-Wallis H test for more than two independent groups. A two-sided test with *P* < 0.05 was regarded as statistically significant, and Stata version 17.0 was utilized for all statistical analyses. STROBE guidelines were followed in this study (38).

## Results

### Study population and characteristics of hospitals

Among 43,963 patients hospitalized for an initial osteoporotic hip fracture between 2013 and 2022, we identified 1,152 patients who experienced a subsequent hip fracture and were re-hospitalized during the study period. Of these, 78.73% were female and 21.27% were male. Additionally, the majority of patients (57.47%) were aged 80 years or older, with 26.13% aged 70–79 years and 16.41% aged 60–69 years. Most patients in this study were married (85.85%), covered by Urban Employee Basic Medical Insurance (69.44%), had third-degree surgery after their first fracture (51.74%), and were discharged on medical advice (regular discharge) (99.57%) (**Table 1**).

**Table 1:** Patient and institutional characteristics associated with hospitalization costs.

Variable	First fracture patients, *n* (%)	First fracture median costs (USD)	*P* value	Second fracture patients, *n* (%)	Second fracture median costs (USD)	*P* value
Gender			0.272			0.206
Male	245 (21.27)	8398.25		245 (21.27)	8404.02	
Female	907 (78.73)	8545.99		907 (78.73)	8791.65	
Age group			0.035			0.407
60~69 years old	189 (16.41)	8552.50		128 (11.11)	9096.53	
70~79 years old	301 (26.13)	8792.78		272 (23.61)	8862.88	
≥80 years old	662 (57.47)	8401.63		752 (65.28)	8637.68	
Marital status			<0.001			0.362
Never married	93 (8.07)	8494.20		41 (3.56)	9045.58	
Married	989 (85.85)	8594.77		1025 (88.98)	8738.16	
Other	70 (6.08)	6701.68		86 (7.47)	7970.17	
Work			<0.001			<0.001
Unemployed	42 (3.65)	8190.50		26 (2.26)	7511.30	
Employed	97 (8.42)	7837.90		97 (8.42)	6879.11	
Retired	593 (51.48)	8844.59		656 (56.94)	8800.95	
Other	420 (36.46)	8477.13		373 (32.38)	8936.58	
Insurance type			<0.01			<0.001
Urban Employee Basic Medical Insurance	800 (69.44)	8683.19		833 (72.31)	8994.45	
Urban-Rural Residents Basic Medical Insurance	228 (19.79)	8057.76		219 (19.01)	7496.00	
Fully self-pay	99 (8.59)	8263.79		58 (5.03)	9715.80	
Other	25 (2.17)	9231.60		42 (3.65)	10033.58	
Surgery level			<0.001			<0.001
Unoperated	76 (6.60)	1147.75		163 (14.15)	1133.36	
First-degree surgery	10 (0.87)	7166.94		24 (2.08)	7300.89	
Second-degree surgery	73 (6.34)	8849.85		35 (3.04)	9020.39	
Third-degree surgery	596 (51.74)	8064.70		521 (45.23)	8679.03	
Fourth-degree surgery	397 (34.46)	9787.15		409 (35.50)	9726.55	
LOS			<0.001			<0.001
≤7 days	239 (20.75)	7169.88		304 (26.39)	7015.68	
8~14 days	449 (38.98)	8336.46		424 (36.81)	8787.81	
15~30 days	402 (34.90)	9591.54		342 (29.69)	9930.67	
≥31 days	62 (5.38)	11197.72		82 (7.12)	11729.95	
Discharge method			<0.01			<0.001
Regular discharge	1147 (99.57)	8516.01		1119 (97.14)	8754.67	
Non-regular discharge	5 (0.43)	1148.11		33 (2.86)	4290.61	
Hospital level			<0.001			<0.001
Primary hospital	27 (2.34)	9297.86		52 (4.51)	5678.63	
Grade-B secondary hospital	484 (42.01)	8240.07		508 (44.10)	8248.05	
Grade-A tertiary hospital	641 (55.64)	8790.47		592 (51.39)	9189.18	
Hospital type			0.967			0.967
Comprehensive hospital	1065 (92.45)	8494.20		1060 (92.01)	8743.78	
Non-comprehensive hospital	87 (7.55)	8955.83		92 (7.99)	7864.39	
Hospital management type			0.201			0.684
Non-profit hospital	1144 (99.31)	8511.12		1130 (98.09)	8694.39	
For-profit hospital	8 (0.69)	7011.24		22 (1.91)	8134.69	

**Figure 2:**
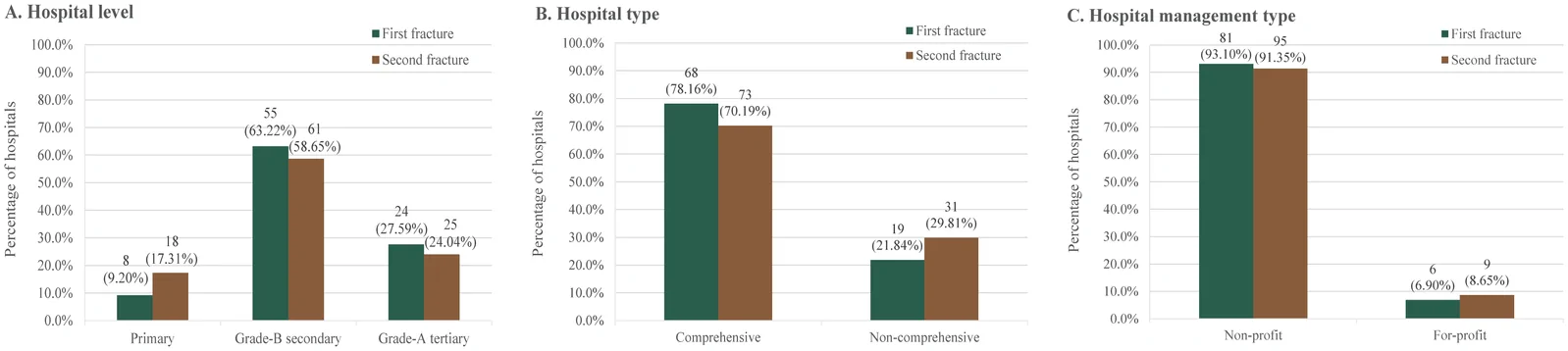
Distribution of hospital characteristics for the first and second fracture hospitalizations **(A)** Hospital level; **(B)** Hospital type; **(C)** Hospital management type. Values above bars indicate the number of hospitals, with percentages in parentheses. Total hospitals for the first fracture hospitalization: 87; for the second fracture hospitalization: 104.

Whether it is the first or second fracture, most patients with subsequent hip fractures were mainly hospitalized in non-profit, comprehensive, and grade-A tertiary hospitals (**Table 1**). However, regarding the number of hospitals, among the 87 hospitals where patients were treated for their first fracture and the 104 hospitals for their second fracture in this study, grade-B secondary hospitals made up the largest share, at 63.22% (for the first fracture) and 58.65% (for the second fracture), respectively. In contrast, grade-A tertiary hospitals comprised 27.59% (*n* = 24) and 24.04% (*n* = 25) of the hospitals for the first and second fractures, respectively, and admitted 641 (55.64%) and 592 (51.39%) of the patients for the respective episodes (**Table 1**; **Figure 2**).

### Univariate analysis of hospitalization costs

Univariate analysis revealed that hospitalization costs for the first fracture differed significantly by age group, marital status, work, insurance type, surgery level, length of stay (LOS), discharge method, and hospital level (all *P* < 0.05). For the second fracture, costs differed significantly by work, insurance type, surgery level, LOS, discharge method, and hospital level (all *P* < 0.001) (**Table 1**). For both episodes, patients undergoing fourth-degree surgery had the highest median costs ($9,787.15 and $9,726.55, respectively), whereas patients without surgery had the lowest median costs ($1,147.75 and $1,133.36). Costs rose substantially with longer LOS, reaching $11,197.72 and $11,729.95 for LOS≥31 days. Insurance type showed significant associations with hospitalization costs in both the first and second fracture episodes. Patients with urban employee basic medical insurance incurred higher costs than those with urban-rural residents basic medical insurance for the first ($8,683.19 vs. $8,057.76) and second ($8,994.45 vs. $7,496.00) fractures.

Cost patterns across hospital levels differed between the first and second fracture episodes. For the first fracture, median costs were higher in grade-A tertiary hospitals than in grade-B secondary hospitals ($8,790.47 vs. $8,240.07), with primary hospitals showing the highest cost ($9,297.86). For the second fracture, grade-A tertiary hospitals also had higher median costs than grade-B secondary hospitals ($9,189.18 vs. $8,248.05), whereas primary hospitals showed the lowest cost ($5,678.63). Differences in age-group distribution were observed only for the first fracture (*P* = 0.035), with patients aged 70~79 years incurring the highest median cost ($8,792.78).

### Multilevel analysis of hospitalization costs for the first fracture

The null model showed that the ICC was 0.333 (95%*CI*, 0.218 to 0.473), with 33.3% of the total variance estimated at the hospital level. The LR test comparing the null model with a single-level linear model was statistically significant (*χ*² = 86.11, *P* < 0.001), with significant clustering of hospitalization costs at the hospital level (**Supplementary Table S9**).

The random intercept model showed that hospitalization costs for the first hip fracture increased with the level of surgery (all *P* < 0.001). On average, patients undergoing fourth-degree surgery had hospitalization costs 7.523 times those of patients who did not undergo surgery (exp(*β*) = 7.523; 95% CI, 6.807 to 8.314; *P* < 0.001). Inpatient costs also rose with longer LOS (all *P* < 0.001); patients staying 31 days or more had costs 2.878 times those with 7 days or fewer (exp(*β*) = 2.878; 95%*CI*, 2.586 to 3.203; *P* < 0.001). Patients treated in grade-A tertiary hospitals had higher costs than those in grade-B secondary hospitals (exp(*β*) = 1.176; 95%*CI*, 1.061 to 1.305; *P* < 0.05), corresponding to a 17.6% increase in hospitalization costs (**Table 2**).

**Table 2:** Multilevel model of factors influencing hospitalization costs for the first fracture in patients with refracture.

Variable	*β* coefficient (95% *CI)*	exp(*β*) (95% *CI*)	*P* value
Age group			
60~69 years old	Reference		
70~79 years old	0.049 (-0.014 to 0.111)	1.050 (0.986 to 1.117)	0.128
≥80 years old	-0.004 (-0.061 to 0.052)	0.996 (0.941 to 1.053)	0.878
Insurance type			
Urban Employee Basic Medical Insurance	Reference		
Urban-Rural Residents Basic Medical Insurance	0.016 (-0.040 to 0.073)	1.016 (0.961 to 1.076)	0.574
Fully self-pay	-0.048 (-0.120 to 0.024)	0.953 (0.887 to 1.024)	0.188
Other	-0.078 (-0.214 to 0.059)	0.925 (0.807 to 1.061)	0.264
Surgery level.			
Unoperated	Reference		
First-degree surgery	1.365 (1.134 to 1.596)	3.916 (3.108 to 4.933)	<0.001
Second-degree surgery	1.715 (1.595 to 1.835)	5.557 (4.928 to 6.265)	<0.001
Third-degree surgery	1.810 (1.715 to 1.904)	6.110 (5.557 to 6.713)	<0.001
Fourth-degree surgery	2.018 (1.918 to 2.118)	7.523 (6.807 to 8.314)	<0.001
LOS			
≤7 days	Reference		
8~14 days	0.264 (0.205 to 0.324)	1.302 (1.228 to 1.383)	<0.001
15~30 days	0.492 (0.423 to 0.561)	1.636 (1.527 to 1.752)	<0.001
≥31 days	1.057 (0.950 to 1.164)	2.878 (2.586 to 3.203)	<0.001
Discharge method			
Regular discharge	Reference		
Non-regular discharge	-0.037 (-0.351 to 0.278)	0.964 (0.704 to 1.320)	0.820
Hospital level			
Grade-B secondary hospital	Reference		
Primary hospital	0.082 (-0.124 to 0.289)	1.085 (0.883 to 1.335)	0.434
Grade-A tertiary hospital	0.162 (0.059 to 0.266)	1.176 (1.061 to 1.305)	<0.05

### Multilevel analysis of hospitalization costs for the second fracture

The null model results indicated an ICC of 0.338 (95%*CI*, 0.230 to 0.467), with 33.8% of the total variance estimated at the hospital level. The LR test confirmed significant between-hospital variation (*χ*² = 69.62, *P* < 0.001) (**Supplementary Table S10**).

The results of the random intercept model revealed that surgery level, LOS, and hospital level were significant factors influencing the cost of hospitalization for the second fracture (all *P* < 0.05). Additionally, compared with an interval of 1 year or less, an interval of 1 to 1.5 years between hospitalizations was associated with significantly lower costs (exp(*β*) = 0.899; 95%*CI*, 0.824 to 0.980; *P* < 0.05). Costs increased progressively with the level of surgery (all *P* < 0.001); fourth-degree surgery was associated with costs 7.783 times those of unoperated patients (exp(*β*) = 7.783; 95%*CI*, 7.043 to 8.601; *P* < 0.001). Regarding LOS, patients hospitalized for 31 days or more had costs 3.254 times those of patients staying 7 days or fewer (exp(*β*) = 3.254; 95%*CI*, 2.861 to 3.702; *P* < 0.001). For hospital level, compared with grade-B secondary hospitals, primary hospitals had significantly higher costs (exp(*β*) = 1.908; 95%*CI*, 1.478 to 2.462; *P* < 0.001), which represents a 90.8% increase, and grade-A tertiary hospitals also had higher costs (exp(*β*) = 1.234; 95%*CI*, 1.024 to 1.487; *P* < 0.05), corresponding to a 23.4% increase (**Table 3**).

**Table 3:** Multilevel model of factors influencing hospitalization costs for the second fracture in patients with refracture.

Variable	*β* coefficient (95% *CI*)	exp(*β*) (95% *CI*)	*P* value
Age group			
60~69 years old	Reference		
70~79 years old	-0.002 (-0.097 to 0.094)	0.998 (0.908 to 1.099)	0.975
≥80 years old	-0.029 (-0.116 to 0.058)	0.971 (0.890 to 1.060)	0.512
Insurance type			
Urban Employee Basic Medical Insurance	Reference		
Urban-Rural Residents Basic Medical Insurance	-0.073 (-0.151 to 0.005)	0.930 (0.860 to 1.005)	0.065
Fully self-pay	-0.071 (-0.192 to 0.051)	0.931 (0.825 to 1.052)	0.256
Other	0.019 (-0.123 to 0.162)	1.019 (0.884 to 1.176)	0.789
Surgery level			
Unoperated	Reference		
First-degree surgery	1.108 (0.907 to 1.310)	3.028 (2.477 to 3.706)	<0.001
Second-degree surgery	1.591 (1.411 to 1.771)	4.909 (4.101 to 5.876)	<0.001
Third-degree surgery	1.914 (1.818 to 2.011)	6.780 (6.160 to 7.463)	<0.001
Fourth-degree surgery	2.052 (1.952 to 2.153)	7.783 (7.043 to 8.601)	<0.001
LOS			
≤7 days	Reference		
8~14 days	0.391 (0.318 to 0.465)	1.478 (1.374 to 1.592)	<0.001
15~30 days	0.613 (0.528 to 0.698)	1.846 (1.696 to 2.010)	<0.001
≥31 days	1.180 (1.051 to 1.309)	3.254 (2.861 to 3.702)	<0.001
Interval between hospitalizations			
≤1 year	Reference		
1~1.5 years	-0.106 (-0.193 to -0.020)	0.899 (0.824 to 0.980)	<0.05
1.5~2 years	-0.031 (-0.118 to 0.056)	0.969 (0.889 to 1.058)	0.483
≥2 years	-0.047 (-0.114 to 0.019)	0.954 (0.892 to 1.019)	0.160
Discharge method			
Regular discharge	Reference		
Non-regular discharge	0.007 (-0.161 to 0.174)	1.007 (0.851 to 1.190)	0.938
Hospital level			
Grade-B secondary hospital	Reference		
Primary hospital	0.646 (0.391 to 0.901)	1.908 (1.478 to 2.462)	<0.001
Grade-A tertiary hospital	0.210 (0.024 to 0.397)	1.234 (1.024 to 1.487)	<0.05

### Multivariable quantile regression of cost differences between two fracture hospitalizations

The hospitalization variations and cost differences between the first and second hospitalizations for osteoporotic hip fractures are shown in **Table 4**. Among the 1,152 patients, the majority showed no variation in hospital level (82.81%) between the two hospitalizations; however, notable shifts were observed for both surgery level and LOS. For surgery level, 31.16% of patients received higher-level surgery and 19.18% received lower-level surgery during the second fracture episode. For LOS, 23.52% of patients had longer LOS and 30.21% had shorter LOS during the second hospitalization. Variations in all three dimensions were significantly associated with cost differences (all *P* < 0.001). The median cost difference was $−83.84 (interquartile range: −$2294.90 to $2239.88).

**Table 4:** Hospitalization variations and cost differences between two hospitalizations.

Variables	Patients		Cost difference (USD)				*χ*²	*P* value
	*N*	%	25th	50th	75th	Mean (SD)		
All	1152	100.0	-2294.90	-83.84	2239.88	-68.19 (5198.61)		
Variation in hospital levels								
No variation	954	82.81	-2113.75	-111.04	2155.06	-109.88 (4687.92)	17.22	<0.001
Higher in the second hospitalization	71	6.16	-1352.58	1497.28	6455.59	2087.03 (5561.87)		
Lower in the second hospitalization	127	11.02	-4765.70	-556.21	1920.30	-959.92 (7748.49)		
Variation in surgery levels								
No variation	572	49.65	-1582.37	-100.05	1854.45	204.64 (3508.28)	20.75	<0.001
Higher in the second hospitalization	359	31.16	-5775.55	-769.34	2555.26	-1103.93 (7080.06)		
Lower in the second hospitalization	221	19.18	-1994.73	536.60	3295.03	908.17 (4997.55)		
Variation in LOS								
No variation	533	46.27	-1559.77	4.28	2069.48	142.81 (4067.54)	130.21	<0.001
Longer in the second hospitalization	271	23.52	-630.24	1487.81	4605.22	2434.64 (5634.35)		
Shorter in the second hospitalization	348	30.21	-5665.91	-1647.52	672.77	-2340.40 (5425.72)		

**Table 5** presents the findings of multivariable quantile regression of hospitalization variation on cost differences at the 25th, 50th, and 75th percentiles. A higher hospital level during the second hospitalization was significantly associated with higher relative costs in the second episode, with coefficients increasing from 2793.54 (95%*CI*, 1138.71 to 4448.37) at the 25th percentile to 3633.86 (95% *CI*, 1758.93 to 5508.78) at the 75th percentile (all *P* < 0.01). Conversely, a lower hospital level was associated with lower relative costs in the second episode across all quantiles, with coefficients of −1790.40 (95% *CI*, −3344.20 to −236.60) at the 25th percentile, −1253.26 (95% *CI*, −2157.43 to −349.10) at the 50th percentile, and −2151.47 (95% *CI*, −3088.86 to −1214.08) at the 75th percentile (all *P* < 0.05).

**Table 5:** Multivariable quantile regression of cost differences between two hospitalizations.

Variable	25th percentile		50th percentile		75th percentile	
	coeff	95% *CI*	coeff	95% *CI*	coeff	95% *CI*
Variation in hospital levels						
No variation	Reference		Reference		Reference	
Higher in the second hospitalization	2793.54	(1138.71 to 4448.37)**	3097.16	(1138.87 to 5055.44)**	3633.86	(1758.93 to 5508.78)***
Lower in the second hospitalization	-1790.40	(-3344.20 to -236.60)*	-1253.26	(-2157.43 to -349.10)**	-2151.47	(-3088.86 to -1214.08)***
Variation in surgery levels						
No variation	Reference		Reference		Reference	
Higher in the second hospitalization	-2967.80	(-3855.67 to -2079.93)***	-960.29	(-1708.51 to -212.09)*	494.68	(-358.21 to 1347.57)
Lower in the second hospitalization	-551.79	(-1378.11 to 274.53)	366.67	(-384.61 to 1117.95)	1354.48	(561.43 to 2147.53)**
Variation in LOS						
No variation	Reference		Reference		Reference	
Longer in the second hospitalization	1391.24	(673.06 to 2109.42)***	1916.18	(1243.60 to 2588.77)***	2780.02	(1866.01 to 3694.03)***
Shorter in the second hospitalization	-3329.95	(-4151.88 to -2508.02)***	-1828.51	(-2558.51 to -1098.51)***	-1573.74	(-2287.27 to -860.20)***

All models were adjusted simultaneously for gender, age group, work, marital status, insurance type, discharge method, and interval between hospitalizations.

***P < 0.001, **P  < 0.01, *P < 0.05

For surgery level, a higher level during the second hospitalization was associated with decreased relative costs in the second episode at the 25th and 50th percentiles (coefficients: −2967.80 and −960.29, respectively; all *P* < 0.05), but not at the 75th percentile. A lower surgery level was significantly associated with higher costs in the second hospitalization relative to the first only at the 75th percentile (coefficient = 1354.48; 95% CI, 561.43 to 2147.53; *P* < 0.01). Regarding LOS, a longer LOS during the second hospitalization was associated with higher relative costs in the second episode across all quantiles, with coefficients ranging from 1391.24 to 2780.02 (all *P* < 0.001). Conversely, a shorter LOS was associated with lower relative costs in the second episode across all quantiles, with the effect attenuating from −3329.95 (95%*CI*, −4151.88 to −2508.02) at the 25th percentile to −1573.74 (95%*CI*, -2287.27 to -860.20) at the 75th percentile (all *P* < 0.001).

## Discussion

This study extends current evidence by separately identifying the factors that influence hospitalization costs for the first and second fracture episodes and further examining the factors associated with cost differences between the two episodes among patients with refractures. Multilevel models were employed to account for random effects at the hospital level, thereby providing a more accurate reflection of the associations. Additionally, multivariable quantile regression was applied to examine cost differences between the two episodes across quantiles, enabling a more nuanced characterization than conventional regression approaches. Our findings revealed that grade-A tertiary hospitals were associated with higher inpatient costs than grade-B secondary hospitals for both fracture episodes. Beyond institutional characteristics, surgery level and LOS were significantly associated with hospitalization costs. When focusing on cost differences between the two hospitalizations, variation in hospital level, surgery level, and LOS showed quantile-varying effects.

Osteoporotic hip fracture patients in Shanghai exhibited a dual distribution pattern: a marked concentration in high-volume grade-A tertiary hospitals, most of which specialized in orthopedics, alongside substantial dispersion across grade-B secondary hospitals. The concentration of grade-A tertiary hospitals reflects the clinical imperative for rapid surgical access and advanced perioperative optimization for frail elderly patients (39–44). Conversely, the dispersion of grade-B secondary hospitals reflects geographic proximity and lower economic barriers for rural and peri-urban populations (2, 36, 45–48). This tension between centralized excellence and local accessibility characterizes hip fracture care under China’s tiered healthcare system (34, 49).

In this study, the null multilevel model revealed that approximately one-third of the total cost variation for both fracture episodes was attributable to between-hospital differences. This clustering is consistent with hospital-level heterogeneity—potentially reflecting differential resource allocation, surgical capacity, treatment protocols, or pricing strategies—and highlights that institutional factors may play a substantial role in shaping inpatient costs alongside patient-level characteristics. The stability of this effect across episodes suggests persistent institutional drivers shaping cost patterns in both first and second hospitalizations. These findings challenge the prevailing patient-centered view of cost determination in prior studies, which have relied predominantly on conventional regression models that assume independent observations and ignore nested data structures (20, 22, 23). By partitioning variance into institutional and individual components, our approach demonstrates that hospital-level factors also contribute to cost variation. This implies that cost-containment policies may need to consider institutional heterogeneity rather than focusing solely on patient characteristics.

Multilevel modeling revealed that grade-A tertiary hospitals incurred significantly higher adjusted costs than grade-B secondary hospitals for both the first and second fractures, with the disparity widening in the second episode. For the second fracture, this finding was robust to changes in sample definition and the choice of reference category: in the initial analytic sample (*n* = 1,267), with primary hospitals as the reference, both grade-B secondary and grade-A tertiary hospitals were significantly less costly than primary hospitals (*β* = −0.724 and −0.491, respectively), with grade-B secondary hospitals appearing to be the least costly setting; in the revised analytic sample (*n* = 1,152), where primary hospital cases were too sparse for reliable estimation, a formal pairwise contrast with grade-B secondary hospitals as the reference confirmed that grade-A tertiary hospitals were significantly more costly than grade-B secondary hospitals, corroborating the non-linear pattern observed in the original analysis. This pattern likely reflects the functional role of grade-A tertiary hospitals as regional referral centers for complex cases, where more intensive resource utilization—including management of multiple comorbidities, unstable fracture patterns, and revision surgeries—resulting in higher adjusted cost estimates than those observed for grade-B secondary hospitals (36, 50). Grade-B secondary hospitals, while maintaining adequate surgical capacity for standard hip fracture procedures, predominantly managed patients with less complicated clinical presentations, thereby achieving lower per-episode costs (36). These findings align with prior investigations showing that high-quality local hospitals can achieve cost containment through efficient use of resources (33, 36, 50–52). The differential effects of hospital levels persisted after adjustment for patient-level confounders, suggesting that unobserved case-mix complexity or institutional-level resource intensity of grade-A tertiary hospitals outweighs the cost-containment capacity observed in secondary settings.

Extending this non-linear pattern to primary hospitals, these hospitals exhibited the highest adjusted costs for the second fracture relative to grade-B secondary and grade-A tertiary hospitals, despite having the lowest descriptive median. Predominantly non-surgical management (69.23%) and prolonged hospitalizations (median LOS = 28 days; IQR, 17.50 to 62.00) (**Supplementary Table S11**) in this subset are consistent with the profile of low-volume centers, where such patterns commonly coincide with higher adjusted costs (53–56). However, with only 52 patients (4.51%) and a markedly skewed LOS distribution indicating extreme outliers, the elevated coefficient likely reflects statistical instability rather than a definitive cost disadvantage.

Multivariable quantile regression of cost differences revealed differential effects of hospital-level changes between episodes. Upgrading to a higher hospital level during the second hospitalization was associated with higher relative costs in the second episode across all quantiles, with the effect most pronounced at the 75th percentile. This may reflect that patients whose hospital level increased were more likely to have more severe conditions or to require more specialized treatment during the second episode (57), resulting in a substantial rise in inter-episode cost disparity. Conversely, downgrading was associated with lower relative costs in the second episode across all quantiles, but the magnitude of this reduction varied across quantiles: the association was more pronounced at the 25th and 75th percentiles and relatively attenuated at the 50th percentile. The attenuated effect at the median suggests that the cost-difference distribution was less responsive to hospital-level downgrades for patients with moderate cost differences, possibly because standardized clinical pathways diluted the cost differential across hospital levels for this subgroup (58, 59).

Collectively, these findings indicate that the relationship between hospital level and costs for osteoporotic hip fracture is context-dependent and varies by fracture episode. While multilevel modeling captured the association between static hospital-level characteristics and costs within each episode, quantile regression further revealed that changes in hospital level—particularly upgrades—were independently related to cost differences between the two episodes. When analyzing the relationship between hospital level and hospitalization costs, a comprehensive assessment should account for both the hospital level at each episode and the variation in hospital level between episodes (60, 61).

Beyond institutional characteristics, surgery level is another major factor associated with costs. The multilevel model results showed that after adjusting for patient- and hospital-level confounders, hospitalization costs for both fracture episodes increased progressively with the surgery level. This pattern aligns with the REDUCE study, which found that the decision to forgo surgery (i.e., not to operate), although rare, associated with cost savings, while routine cementation of arthroplasties was linked to higher costs (23). Likewise, a local study in Shanghai demonstrated that hospitalization costs among hip fracture patients escalated with surgical complexity (43). Several mechanisms may underlie this gradient. High-complexity surgeries—particularly third- and fourth-degree surgeries—require specialized equipment, skilled personnel, and increased anesthesia support, all of which increase direct medical costs (37). Moreover, complicated operations necessitate more extensive preoperative workup and longer preparation time, further raising expenditures (62). Due to their extremely high risks, high-level surgeries often necessitate multidisciplinary consultations and comprehensive risk assessments, all of which add to the overall costs (63, 64). While these mechanisms are clinically plausible, it should be noted that the surgery level may partly reflect underlying clinical severity and treatment complexity rather than being a purely independent determinant of hospitalization costs (65). Because our administrative data lack detailed measures of clinical severity (e.g., ICU use), this variable may partially mediate the effect of unobserved disease burden. Therefore, it should be interpreted as a process-of-care indicator correlated with clinical severity, and its association with costs does not imply an independent causal effect.

When shifting from hospitalization costs for each fracture episode to cost differences between two fracture hospitalizations, multivariable quantile regression revealed heterogeneous associations between variation in surgery levels and cost differences across quantiles. At the 25th and 50th percentiles, a higher surgery level in the second episode was associated with lower relative costs, whereas at the 75th percentile, this association was no longer observed; instead, a lower surgery level was significantly associated with higher relative costs. These heterogeneous patterns suggest that the economic impact of surgery-level change is not uniform but depends on the patient’s position in the cost-difference distribution, possibly reflecting differential interactions among surgical intensity, perioperative complexity, and underlying clinical severity (66, 67).

Consistent with the univariate findings, multilevel model results demonstrated a positive association between LOS and inpatient costs in both fracture episodes, with longer LOS corresponding to substantially higher costs, partly because daily charges accumulate over time. This is consistent with findings from a Swiss insurance claims analysis of hospitalized fracture patients, which demonstrated substantial direct economic burden associated with hospitalization and identified LOS as a key cost driver (68). Regarding cost differences between episodes, multivariable quantile regression results showed that a longer LOS during the second hospitalization was associated with higher relative costs in the second episode across all quantiles, with the effect intensifying toward the upper tail (75th quantile). Conversely, a shorter LOS was associated with lower relative costs in the second episode across all quantiles, though the magnitude of this reduction was most pronounced at the lower tail (25th quantile) and progressively attenuated toward the upper tail. These opposing quantile-specific trends suggest that the cost impact of LOS variation is not uniform across the conditional distribution.

Nevertheless, these findings do not suggest that extending the LOS directly increases costs or that shortening it would automatically reduce costs. LOS is influenced by clinical factors (e.g., fracture severity, postoperative complications), institutional practices (e.g., discharge planning), and unmeasured patient conditions (69–71). The LOS serves as a composite indicator of clinical complexity and resource consumption within this associational framework. Residual confounding is likely, and LOS may function as a mediator or collider in the cost-generating process. Future studies using causal mediation analysis or structural equation modeling may further disentangle the direct, indirect, and confounded pathways linking LOS to inpatient costs (72).

This study has several limitations. First, as this analysis used only the front pages of inpatient medical records, which lacked fracture laterality and imaging confirmation the outcome should be interpreted as “subsequent hospitalization for hip fracture” rather than strictly as “new or contralateral hip fracture”. Sensitivity analyses with alternative washout periods were not conducted due to model complexity. The 180-day window was selected as the primary criterion because it is the standard approach in relative research and aligns with the typical rehabilitation and complication-resolution timeline (73–76). A 90-day window would likely capture more readmissions, implant-related complications, and delayed treatment episodes rather than true incident fractures, whereas a 365-day window could be affected by competing risks (e.g., mortality). Future studies should examine multiple washout windows (e.g., 90 and 365 days) to validate the robustness of the 180-day definition. Second, this study did not apply competing-risk analysis to second fracture incidence. Because the primary aim was to identify cost determinants among patients who had already been hospitalized for a second hip fracture, rather than to estimate the risk of a second fracture, competing-risk methods were applied. However, mortality after hip fracture is high and strongly competes with the occurrence of a second fracture. Future studies focused on recurrence risk should employ competing-risk frameworks (e.g., Fine-Gray models or cumulative incidence functions with death as a competing event). Third, despite the use of a stepwise selected multilevel model, residuals at the individual level still existed, thereby affecting the validity of statistical inference. Fourth, due to reliance on the front pages of inpatient medical records, this study was unable to capture detailed clinical information (e.g., implant type, procedure complexity) and socioeconomic indicators (e.g., income, education), nor could it fully adjust for comorbidity burden, treatment patterns, survival status or other hospital- and patient-level factors such as surgical volume, referral pathways, and postoperative outcomes. These unmeasured factors may confound the associations between patient characteristics (including age) and hospitalization costs. Consequently, these associations should be interpreted as observational relationships rather than intrinsic determinants. Moreover, the multilevel cost determinant analyses were limited to patients who experienced a second hip fracture. Therefore, the first-fracture cost profile reflects only the initial hospitalization costs of patients who eventually refracture, not all patients with an index osteoporotic hip fracture. This may introduce selection bias and limit the generalizability of the cost findings. However, this high-burden group is the priority target for secondary prevention and incurs substantial cumulative medical costs. Understanding its cost burden and determinants remains clinically important. Future studies should include all patients with an initial hip fracture to compare cost drivers between refracture and non-refracture groups.

## Conclusion

Hospital-level clustering was present among patients with subsequent osteoporotic hip fractures, accounting for approximately one-third of the variance in hospitalization costs. Grade-A tertiary hospitals incurred significantly higher adjusted costs than grade-B secondary hospitals for both episodes, with the disparity widening in the second episode. Higher surgery level and longer LOS were positively correlated with costs. A higher hospital level and longer LOS during the second hospitalization were associated with higher relative costs in the second episode, whereas higher surgery level was associated with lower relative costs, an association observed only at the 25th and 50th percentiles. These findings highlight the importance of developing episode-specific cost-control strategies for subsequent fractures that are tailored to each episode, while also accounting for variations between the two hospitalizations.

## Data Availability

The datasets generated and analyzed during the current study are not publicly available due to institutional policy restrictions governing sensitive medical and financial data. Requests to access the datasets should be directed to yanjing@shdrc.org.
