# Supplementary material for: Influencing factors of hospitalization costs for patients with subsequent osteoporotic hip fractures: multilevel model analysis by fracture episode
Source: Front Endocrinol (Lausanne). 2026 Aug 24;17:1853745. doi: 10.3389/fendo.2026.1853745 (PMC13547062; doi:10.3389/fendo.2026.1853745)
Supplement: Supplementary file 1 [file DataSheet1.docx]

**Appendix and Supplementary Materials**

| **Table S1** **Inflation Adjustment of Hospitalization Costs (2013–2022) Using the Healthcare Consumer Price Index** | | | |
| --- | --- | --- | --- |
| Year | CPI/% | Unadjusted Cost | Adjusted cost |
| 2013 | 100.0 | Y1 | Y1'=Y1*102.1%*98.9%*101.2%*103.3%*102.4%*106.6%*109%*99.3%*100.4% |
| 2014 | 100.4 | Y2 | Y2'=Y2*102.1%*98.9%*101.2%*103.3%*102.4%*106.6%*109%*99.3% |
| 2015 | 99.3 | Y3 | Y3'=Y3*102.1%*98.9%*101.2%*103.3%*102.4%*106.6%*109% |
| 2016 | 109.0 | Y4 | Y4'=Y4*102.1%*98.9%*101.2%*103.3%*102.4%*106.6% |
| 2017 | 106.6 | Y5 | Y5'=Y5*102.1%*98.9%*101.2%*103.3%*102.4% |
| 2018 | 102.4 | Y6 | Y6'=Y6*102.1%*98.9%*101.2%*103.3% |
| 2019 | 103.3 | Y7 | Y7'=Y7*102.1%*98.9%*101.2% |
| 2020 | 101.2 | Y8 | Y8'=Y8*102.1%*98.9% |
| 2021 | 98.9 | Y9 | Y9'=Y9*102.1% |
| 2022 | 102.1 | Y10 | Y10'=Y10 |

Note: The cumulative adjustment factor represents the product of chain-linked healthcare CPI ratios from the index year to 2022. Adjusted cost = Unadjusted cost × Cumulative adjustment factor. CPI data were sourced from the Shanghai Statistical Yearbook (Shanghai Municipal Bureau of Statistics).

| **Table S2 Patient and institutional characteristics associated with hospitalization costs (CNY)** | | | | | | |
| --- | --- | --- | --- | --- | --- | --- |
| Variable | First fracture patients, *n* (%) | First fracture median costs (CNY) | *P* value | Second fracture patients, *n* (%) | Second fracture median costs (CNY) | *P* value |
| Gender |  |  | 0.272 |  |  | 0.206 |
| Male | 245 (21.27) | 56487.47 |  | 245 (21.27) | 56526.28 |  |
| Female | 907 (78.73) | 57481.18 |  | 907 (78.73) | 59133.52 |  |
| Age group |  |  | 0.035 |  |  | 0.407 |
| 60~69 years old | 189 (16.41) | 57524.97 |  | 128 (11.11) | 61184.17 |  |
| 70~79 years old | 301 (26.13) | 59141.12 |  | 272 (23.61) | 59612.62 |  |
| ≥80 years old | 662 (57.47) | 56510.2 |  | 752 (65.28) | 58097.9 |  |
| Marital status |  |  | <0.001 |  |  | 0.362 |
| Never married | 93 (8.07) | 57132.84 |  | 41 (3.56) | 60841.48 |  |
| Married | 989 (85.85) | 57809.28 |  | 1025 (88.98) | 58773.74 |  |
| Other | 70 (6.08) | 45076.17 |  | 86 (7.47) | 53608.16 |  |
| Work |  |  | <0.001 |  |  | <0.001 |
| Unemployed | 42 (3.65) | 55090.12 |  | 26 (2.26) | 50521.75 |  |
| Employed | 97 (8.42) | 52718.5 |  | 97 (8.42) | 46269.58 |  |
| Retired | 593 (51.48) | 59489.6 |  | 656 (56.94) | 59196.07 |  |
| Other | 420 (36.46) | 57018.02 |  | 373 (32.38) | 60108.33 |  |
| Insurance type |  |  | <0.01 |  |  | <0.001 |
| Urban Employee Basic Medical Insurance | 800 (69.44) | 58404 |  | 833 (72.31) | 60497.57 |  |
| Urban-Rural Residents Basic Medical Insurance | 228 (19.79) | 54197.3 |  | 219 (19.01) | 50418.85 |  |
| Fully self-pay | 99 (8.59) | 55583.08 |  | 58 (5.03) | 65349.44 |  |
| Other | 25 (2.17) | 62092.66 |  | 42 (3.65) | 67486.86 |  |
| Surgery level |  |  | <0.001 |  |  | <0.001 |
| Unoperated | 76 (6.60) | 7719.88 |  | 163 (14.15) | 7623.09 |  |
| First-degree surgery | 10 (0.87) | 48205.56 |  | 24 (2.08) | 49106.52 |  |
| Second-degree surgery | 73 (6.34) | 59524.98 |  | 35 (3.04) | 60672.05 |  |
| Third-degree surgery | 596 (51.74) | 54243.98 |  | 521 (45.23) | 58376.02 |  |
| Fourth-degree surgery | 397 (34.46) | 65829.35 |  | 409 (35.50) | 65421.75 |  |
| Length of stay |  |  | <0.001 |  |  | <0.001 |
| ≤7 days | 239 (20.75) | 48225.33 |  | 304 (26.39) | 47188.17 |  |
| 8~14 days | 449 (38.98) | 56071.86 |  | 424 (36.81) | 59107.69 |  |
| 15~30 days | 402 (34.90) | 64513.66 |  | 342 (29.69) | 66794.68 |  |
| ≥31 days | 62 (5.38) | 75316.98 |  | 82 (7.12) | 78896.82 |  |
| Discharge method |  |  | <0.01 |  |  | <0.001 |
| Regular discharge | 1147 (99.57) | 57279.53 |  | 1119 (97.14) | 58884.79 |  |
| Non-regular discharge | 5 (0.43) | 7722.3 |  | 33 (2.86) | 28859.07 |  |
| Hospital level |  |  | <0.001 |  |  | <0.001 |
| Primary hospital | 27 (2.34) | 62538.34 |  | 52 (4.51) | 38195.03 |  |
| Grade-B secondary hospital | 484 (42.01) | 55423.53 |  | 508 (44.10) | 55477.21 |  |
| Grade-A tertiary hospital | 641 (55.64) | 59125.58 |  | 592 (51.39) | 61807.34 |  |
| Hospital type |  |  | 0.967 |  |  | 0.967 |
| Comprehensive hospital | 1065 (92.45) | 57132.84 |  | 1060 (92.01) | 58811.54 |  |
| Non-comprehensive hospital | 87 (7.55) | 60237.81 |  | 92 (7.99) | 52896.67 |  |
| Hospital management type |  |  | 0.201 |  |  | 0.684 |
| Non-profit hospital | 1144 (99.31) | 57246.64 |  | 1130 (98.09) | 58479.34 |  |
| For-profit hospital | 8 (0.69) | 47158.3 |  | 22 (1.91) | 54714.74 |  |

| **Table S3 Hospitalization variations and cost differences between two osteoporotic hip fractures (CNY)** | | | | | | | | |
| --- | --- | --- | --- | --- | --- | --- | --- | --- |
| Variables | Patients | | Cost difference (CNY) | | | | *χ*² | *P* value |
|  | *N* | % | 25th | 50th | 75th | Mean (SD) |  |  |
| All | 1152 | 100 | -15435.73 | -563.92 | 15065.66 | -458.65 (34966.37) |  |  |
| Variation in hospital levels |  |  |  |  |  |  |  |  |
| No variation | 954 | 82.81 | -14217.29 | -746.87 | 14495.15 | -739.06 (31531.42) | 17.22 | <0.001 |
| Higher in the second hospitalization | 71 | 6.16 | -9097.59 | 10070.86 | 43420.94 | 14037.57 (37409.69) |  |  |
| Lower in the second hospitalization | 127 | 11.02 | -32054.57 | -3741.12 | 12916.13 | -6456.52 (52117.12) |  |  |
| Variation in surgery levels |  |  |  |  |  |  |  |  |
| No variation | 572 | 49.65 | -10643.18 | -672.95 | 12473.22 | 1376.43 (23597.04) | 20.75 | <0.001 |
| Higher in the second hospitalization | 359 | 31.16 | -38846.93 | -5174.66 | 17186.93 | -7425.14 (47621.19) |  |  |
| Lower in the second hospitalization | 221 | 19.18 | -13416.75 | 3609.23 | 22162.7 | 6108.44 (33614.02) |  |  |
| Variation in length of stay |  |  |  |  |  |  |  |  |
| No variation | 533 | 46.27 | -10491.17 | 28.79 | 13919.53 | 960.55 (27358.68) | 130.21 | <0.001 |
| Higher in the second hospitalization | 271 | 23.52 | -4239.06 | 10007.16 | 30975.17 | 16375.63 (37897.2) |  |  |
| Lower in the second hospitalization | 348 | 30.21 | -38109.48 | -11081.38 | 4525.12 | -15741.76 (36493.94) |  |  |

| **Table S4 Multivariable quantile regression of cost differences between two hospitalizations (CNY)** | | | | | | |
| --- | --- | --- | --- | --- | --- | --- |
| Variable | 25th percentile | | 50th percentile | | 75th percentile | |
|  | coeff | 95%*CI* | coeff | 95%*CI* | coeff | 95%*CI* |
| Variation in hospital levels | |  |  |  |  |  |
| No variation | Reference | | Reference | | Reference | |
| Higher in the second hospitalization | 18789.63 | (7659.08 to 29920.18)** | 20831.81 | (7660.15 to 34003.39)** | 24441.71 | (11830.74 to 37052.61)*** |
| Lower in the second hospitalization | -12042.41 | (-22493.42 to -1591.4)* | -8429.55 | (-14511.09 to -2348.08)** | -14471.00 | (-20775.98 to -8166.02)*** |
| Variation in surgery levels | |  |  |  |  |  |
| No variation | Reference | | Reference | | Reference | |
| Higher in the second hospitalization | -19961.72 | (-25933.62 to -13989.82)*** | -6459.01 | (-11491.61 to -1426.54)* | 3327.27 | (-2409.36 to 9063.89) |
| Lower in the second hospitalization | -3711.39 | (-9269.31 to 1846.52) | 2466.26 | (-2586.93 to 7519.44) | 9110.37 | (3776.23 to 14444.50)** |
| Variation in length of stay | |  |  |  |  |  |
| No variation | Reference | | Reference | | Reference | |
| Longer in the second hospitalization | 9357.62 | (4527.07 to 14188.17)*** | 12888.42 | (8364.58 to 17412.33)*** | 18698.69 | (12550.97 to 24846.42)*** |
| Shorter in the second hospitalization | -22397.58 | (-27925.96 to -16869.19)*** | -12298.74 | (-17208.79 to -7388.69)*** | -10585.13 | (-15384.41 to -5785.79)*** |
| Note: All models were adjusted simultaneously for gender, age group, work, marital status, insurance type, discharge method, and interval between hospitalizations. | | | | | | |
| *** *P* < 0.001, ** *P*  < 0.01, * *P* < 0.05 | | | | | | |

| **Table S5 Model-building results for multilevel analyses of the first fracture hospitalization costs** | | | | | | | | | | | |  |
| --- | --- | --- | --- | --- | --- | --- | --- | --- | --- | --- | --- | --- |
| Model | N | df | ll(model) | AIC | BIC | Variables included | Compared to | LR *χ*² | df (LR) | *P* value | Decision |  |
| null | 1152 | 3 | -1072.224 | 2150.448 | 2165.596 | Random intercept only | — | — | — | — | — |  |
| L1 | 1152 | 22 | -411.998 | 867.996 | 979.08 | Age + Insurance + Surgery + LOS + Discharge + Gender + Marriage + Work | null | 1320.45 | 19 | <0.001 | **Retain** |  |
| reduced_gender | 1152 | 21 | -412.663 | 867.325 | 973.36 | Age + Insurance + Surgery + LOS + Discharge + Marriage + Work | L1 | 1.33 | 1 | 0.249 | **Remove** |  |
| reduced_marriage | 1152 | 19 | -413.467 | 864.934 | 960.87 | Age + Insurance + Surgery + LOS + Discharge + Work | reduced_gender | 1.61 | 2 | 0.447 | **Remove** |  |
| reduced_work | 1152 | 16 | -414.664 | 861.327 | 942.115 | Age + Insurance + Surgery + LOS + Discharge | reduced_marriage | 2.39 | 3 | 0.494 | **Remove** |  |
| L2 | 1152 | 20 | -409.695 | 859.39 | 960.376 | Age + Insurance + Surgery + LOS + Discharge + Hospital level + Type + Management type | reduced_work | 9.94 | 4 | 0.042 | **Retain** |  |
| reduced_hosp_type | 1152 | 19 | -410.177 | 858.354 | 954.289 | Age + Insurance + Surgery + LOS + Discharge + Hospital level + Management type | L2 | 0.96 | 1 | 0.326 | **Remove** |  |
| **reduced_hosp_type_2** | **1152** | **18** | **-410.288** | **856.576** | **947.463** | **Age + Insurance + Surgery + LOS + Discharge + Hospital level** | **reduced_hosp_type** | **0.22** | **1** | **0.637** | **Final** |  |
| Note: All models were estimated by maximum likelihood (ML) to ensure valid likelihood-ratio comparisons. The final model (reduced_hosp_type_2) achieves the lowest AIC (856.576) and a near-optimal BIC (947.463), retaining hospital level as the sole Level-2 predictor. LR tests confirm that removing gender, marital status, and work did not significantly degrade model fit (all *p* > 0.05). The addition of hospital-level variables significantly improved model fit (*p* = 0.042), but hospital type and hospital management type were individually non-significant (*p* = 0.326 and *p* = 0.637, respectively). | | | | | | | | | | | |  |
|  |  |  |  |  |  |  |  |  |  |  |  |  |

| **Table S6 Model-building results for multilevel analyses of the second fracture hospitalization costs** | | | | | | | | | | | |  |
| --- | --- | --- | --- | --- | --- | --- | --- | --- | --- | --- | --- | --- |
| Model | N | df | ll(model) | AIC | BIC | Variables included | Compared to | LR *χ*² | df (LR) | *P* value | Decision |  |
| null | 1152 | 3 | -1479.597 | 2965.194 | 2980.342 | Random intercept only | — | — | — | — | — |  |
| L1 | 1152 | 25 | -758.524 | 1567.049 | 1693.28 | Age + Insurance + Surgery + LOS + Interval + Discharge + Gender + Marriage + Work | null | 1442.2 | 22 | <0.001 | **Retain** |  |
| reduced_gender | 1152 | 24 | -759.275 | 1566.55 | 1687.732 | Age + Insurance + Surgery + LOS + Interval + Discharge + Marriage + Work | L1 | 1.5 | 1 | 0.221 | **Remove** |  |
| reduced_marriage | 1152 | 22 | -760.716 | 1565.432 | 1676.515 | Age + Insurance + Surgery + LOS + Interval + Discharge + Work | reduced_gender | 2.88 | 2 | 0.237 | **Remove** |  |
| reduced_work | 1152 | 19 | -762.489 | 1562.978 | 1658.914 | Age + Insurance + Surgery + LOS + Interval + Discharge | reduced_marriage | 3.55 | 3 | 0.315 | **Remove** |  |
| L2 | 1152 | 23 | -748.319 | 1542.639 | 1658.772 | Age + Insurance + Surgery + LOS + Interval + Discharge + Hospital level + Type + Management type | reduced_work | 28.34 | 4 | <0.001 | **Retain** |  |
| reduced_hosp_type | 1152 | 22 | -750.126 | 1544.253 | 1655.336 | Age + Insurance + Surgery + LOS + Interval + Discharge + Hospital level + Management type | L2 | 3.61 | 1 | 0.057 | **Remove** |  |
| **reduced_hosp_type_2** | **1152** | **21** | **-750.193** | **1542.386** | **1648.420** | **Age + Insurance + Surgery + LOS + Interval + Discharge + Hospital level** | **reduced_hosp_type** | **0.13** | **1** | **0.715** | **Final** |  |
| Note: All models were estimated by maximum likelihood (ML) to ensure valid likelihood-ratio comparisons. The final model (reduced_hosp_type_2) achieves both the lowest AIC (1542.386) and the lowest BIC (1648.420). LR tests confirm that removing gender, marital status, and work did not significantly degrade model fit (all *p* > 0.05). The addition of hospital-level variables significantly improved model fit (*p* <0.001), but hospital type and hospital management type were individually non-significant (*p* = 0.057 and *p* = 0.715, respectively). | | | | | | | | | | | |  |
|  |  |  |  |  |  |  |  |  |  |  |  |  |

**Table S7 Multicollinearity diagnostics for the first and second fracture multilevel models**

| Variable | First fracture VIF | Second fracture VIF |
| --- | --- | --- |
| Age group |  |  |
| 60~69 years old | Reference | |
| 70~79 years old | 1.98 | 2.46 |
| ≥80 years old | 2 | 2.52 |
| Insurance type |  |  |
| Urban Employee Basic Medical Insurance | Reference | |
| Urban-Rural Residents Basic Medical Insurance | 1.11 | 1.15 |
| Fully self-pay | 1.06 | 1.07 |
| Other | 1.02 | 1.03 |
| Surgery level |  |  |
| Unoperated | Reference | |
| First-degree surgery | 1.16 | 1.16 |
| Second-degree surgery | 2.01 | 1.26 |
| Third-degree surgery | 5.27 | 3.01 |
| Fourth-degree surgery | 5.12 | 2.91 |
| Length of stay |  |  |
| ≤7 days | Reference | |
| 8~14 days | 1.84 | 1.67 |
| 15~30 days | 2.04 | 1.79 |
| ≥31 days | 1.29 | 1.37 |
| Interval between hospitalizations |  |  |
| ≤1 year | Reference | |
| 1~1.5 years | — | 1.42 |
| 1.5~2 years | — | 1.42 |
| ≥2 years | — | 1.63 |
| Discharge method |  |  |
| Regular discharge | Reference | |
| Non-regular discharge | 1.09 | 1.08 |
| Hospital level |  |  |
| Grade-B secondary hospital | Reference | |
| Primary hospital | 1.14 | 1.32 |
| Grade-A tertiary hospital | 1.29 | 1.29 |
| Model Summary |  |  |
| Mean VIF | 1.96 | 1.64 |
| Condition number | 13.81 | 12.16 |
| Note: VIF = variance inflation factor. Reference categories are indicated by Reference. The condition number was calculated from the scaled raw sum-of-squares and cross-products matrix. | | |

| **Table S8 Generalized linear mixed model of factors influencing hospitalization costs for the first fracture in patients with refracture** | | |
| --- | --- | --- |
| Parameter | *β* coefficient (*95%CI)* | *P* value |
| Age group |  |  |
| 60~69 years old | Reference | |
| 70~79 years old | 0.055 (-0.012 to 0.123) | 0.106 |
| ≥80 years old | -0.019 (-0.070 to 0.033) | 0.478 |
| Insurance type |  |  |
| Urban Employee Basic Medical Insurance | Reference | |
| Urban-Rural Residents Basic Medical Insurance | -0.008 (-0.053 to 0.038) | 0.744 |
| Fully self-pay | -0.057 (-0.133 to 0.018) | 0.135 |
| Other | -0.068 (-0.228 to 0.091) | 0.399 |
| Surgery level |  |  |
| Unoperated | Reference | |
| First-degree surgery | 1.415 (0.829 to 2.001) | <0.001 |
| Second-degree surgery | 1.488 (1.186 to 1.791) | <0.001 |
| Third-degree surgery | 1.552 (1.301 to 1.803) | <0.001 |
| Fourth-degree surgery | 1.763 (1.502 to 2.024) | <0.001 |
| Length of stay |  |  |
| ≤7 days | Reference | |
| 8~14 days | 0.255 (0.180 to 0.330) | <0.001 |
| 15~30 days | 0.461 (0.374 to 0.548) | <0.001 |
| ≥31 days | 1.102 (0.850 to 1.355) | <0.001 |
| Discharge method |  |  |
| Regular discharge | Reference | |
| Non-regular discharge | -0.234 (-0.704 to 0.235) | 0.328 |
| Hospital level |  |  |
| Grade-B secondary hospital | Reference | |
| Primary hospital | 0.082 (-0.200 to 0.363) | 0.571 |
| Grade-A tertiary hospital | 0.186 (0.088 to 0.284) | <0.001 |

| **Table S9 Null model of hospitalization costs for the first fracture***† | | | | |
| --- | --- | --- | --- | --- |
| Effect | Parameter | Estimate | Standard error | 95%*CI* |
| Fixed effect |  |  |  |  |
| Intercept | $\beta_{0}$ | 10.779 | 0.052 | (10.676 to 10.881) |
| Random effect |  |  |  |  |
| Level 2 (hospital) | $\sigma_{u_{0}}^{2}$ | 0.167 | 0.048 | (0.095 to 0.293) |
| Level 1 (patient) | $\sigma_{e_{0}}^{2}$ | 0.335 | 0.015 | (0.307 to 0.365) |
| *LR test vs. linear model: *χ*^2^ = 86.11, *P* < 0.001  †ICC = 0.333 (0.066), 95% *CI*: 0.218 to 0.473 | | |  |  |

| **Table S10 Null model of hospitalization costs for the second fracture*†** | | | | |
| --- | --- | --- | --- | --- |
| Effect | Parameter | Estimate | Standard error | 95%*CI* |
| Fixed effect |  |  |  |  |
| Intercept | $\beta_{0}$ | 10.562 | 0.070 | (10.425 to 10.699) |
| Random effect |  | | | |
| Level 2 (hospital) | $\sigma_{u_{0}}^{2}$ | 0.343 | 0.089 | (0.206 to 0.571) |
| Level 1 (patient) | $\sigma_{e_{0}}^{2}$ | 0.670 | 0.030 | (0.614 to 0.732) |
| *LR test vs. linear model: *χ*^2^ = 69.62, *P* < 0.001  †ICC = 0.338 (0.061), 95% *CI*: 0.230 to 0.467 | | |  |  |

| **Table S11 Characteristics of the second fracture episode by hospital level** | | | |
| --- | --- | --- | --- |
| Characteristic | Primary hospital (n = 52) | Grade-B secondary hospital (n = 508) | Grade-A tertiary hospital (n = 592) |
| Length of stay, days |  |  |  |
| Mean (SD) | 57.44 (76.19) | 15.69 (12.39) | 12.68 (12.80) |
| Median (IQR) | 28.00 (17.50–62.00) | 13.00 (8.00–19.00) | 9.00 (7.00–15.00) |
| Hospitalization cost, USD |  |  |  |
| Mean (SD) | 8475.09 (9348.06) | 7850.00 (4043.64) | 9431.46 (4195.50) |
| Median (IQR) | 5678.63 (2265.49–12100.74) | 8248.05 (5692.83–10482.93) | 9189.18 (7250.20–11462.10) |
| Surgical management |  |  |  |
| Non-surgical, *n* (%) | 36 (69.23) | 82 (16.14) | 45 (7.60) |
| Surgical, *n* (%) | 16 (30.77) | 426 (83.86) | 547 (92.40) |

**Figure S1 Multilevel model assumption diagnostics.** (A) Level-1 residual Q-Q plots; (B) Level-1 residuals versus linear predictor; (C) Level-1 residual distributions; (D) Level-2 random intercept Q-Q plots; (E) Level-2 random intercept distributions. In each panel, the left and right subplots correspond to the first and second fracture models, respectively.

**Figure S2 Diagnostic comparison of the multilevel model (MLM) on log costs (left) and the generalized linear mixed model (GLMM) with gamma distribution and log link (right).** (A) Residuals versus linear predictor; (B) normal Q–Q plots; (C) residual distributions; (D) hospital-level random intercept distributions.
